# Brachycephalics: *‘Once a problem is seen it cannot be unseen’*

**DOI:** 10.1186/s40575-023-00126-z

**Published:** 2023-04-07

**Authors:** Brenda N. Bonnett

**Affiliations:** B Bonnett Consulting, Wiarton, ON Canada

Most dog breeds we see today have been generated over the last two to three hundred years. These have been ‘created’ through human intervention of selecting dogs who exhibit particular physical or behavioural characteristics. Such traits have then been genetically fixed through inbreeding and back-crossing. This has resulted in highly characteristic inbred lines of dogs we call pedigrees. Each pedigree exhibits varyingly a high level of consanguinity (i.e. level of shared genetic identity) and in some breeds this is seen at extreme levels. Homogeneity and retention of desired breed characteristics have been maintained through two related mechanisms. The first is the formation of national pedigree dog’s registration systems (usually national Kennel Clubs); the second is related to the first and was the development of agreed and standardised breed characteristics and parameters. These mechanisms have been largely catalysed by the formation of specific breed clubs and high profile and competitive pedigree dog shows.

An inadvertent and unintended consequence of the high levels of inbreeding in many dog breeds is that some other variants of genes which cause significant diseases, or extreme anatomical or physiological issues have also been ‘trapped’ within the breed development. The accolades of champion dogs which classically typify breed standards and the frequent use of preferred sires for breeding can further drive reduced genetic heterogeneity within breeds as well as potentially increase in deleterious gene frequencies. We now know that some important dog genetic diseases are due to a single gene and often autosomal recessive conditions (i.e. the dog must have inherited a disease-causing version of the gene from each parent). These have been more straightforward to tackle so long as a genetic test can be developed. If so, genetic screening can then be introduced which can be utilised in selective breeding programmes to reduce the disease gene frequency in the breed. In contrast we now know that many important health conditions, especially those relating to extreme or exaggerated anatomical features, are much more complex and may be a consequence of information encoded in many different genes.

Across the large number of pedigree dogs recognised today, it is clear that some breeds are much more popular than others. Fashions come and go and consequently the number of dogs in a breed population will dynamically change over time. This, in itself, causes two problems. If a breed population size crashes to being very small, it will cause a ‘genetic bottle-neck’ where the size and diversity of the gene pool will become very small. The problem for brachycephalic breeds is the opposite one: when a breed expands in population size very quickly this may be driven by relatively restricted numbers of breeding animals which bring along inadvertent health issues not fully recognised before and/or magnifies existing problems of health and welfare. These issues are enhanced when popularity is driven by the same conformational traits that are associated with poor health or welfare.

To address a problem it has to be first defined. This should be evidence-based, preferably generated from representative large data sets and analysed robustly and rigorously. Unfortunately canine medicine and research has only relatively recently embarked on collecting large longitudinal datasets through epidemiological studies and comprehensive analysis of insurance data. Previously much knowledge has only been based on case series and reports. Now more robust and informative data is now being generated it is critical to establish how such information should be used to improve dog health and well-being. This will require a far more collaborative effort across scientists, kennel clubs, breed clubs, owners, and breeders. Notwithstanding these facts, there are sometimes obvious problems which can/need to be defined and understood to a great degree based on observation and expert opinion.

The Journal of Canine Medicine and Genetics has been a pioneer in publishing epidemiological based studies relating to the health conditions affecting a wide range of breeds [1]. Of these, the data collected through VetCompass have been highly informative [2]. One of the complex health issues now generating major problems for dogs, owners and veterinarians are airway and other conditions related to brachycephalic dogs. In two recent papers published by this Journal, Dan O’Neill and co-authors examine the health of Pug dogs [3] and English Bulldogs in the UK [4] and provide epidemiological evidence and important insights into disease predisposition.

Pug dogs are more likely to suffer from breathing, eye and skin disorders when compared to other dog breeds. Pugs were at 53.92 times greater risk of having brachycephalic obstructive airway syndrome (occurring in 6.6% of Pugs but 0.1% of non-Pugs), and 51.25 times greater risk of stenotic nares or narrow nostrils (occurring in 2.7% of Pugs but 0.0% of other dogs). Similarly Pugs were also at 13 times greater risk of corneal ulceration and 11 times greater risk of skin fold dermatitis when compared to all other breeds. This information is particularly worrying as this and other brachycephalic breeds (French Bulldog and English Bulldog) have been rapidly increasing in popularity, together accounting for over 1/5^th^ of newly registered dogs in The KC [5].

It was therefore no surprise to read soon after the study on Pugs that significant health conditions currently exist in the UK for English Bulldogs [4]. From analysing a random sample of 2,662 English Bulldogs and a control sample of approximately 5 times the size, it was apparent that they had significantly and much high risk of skin fold dermatitis, prolapsed nictitating membrane gland and mandibular prognathism. Furthermore there was increased risks for developing lower respiratory tract disorders and tail disorders. The authors conclude that the health of English Bulldogs is substantially lower than other breeds and that this is likely to be driven by the extreme phenotypic conformation of the English Bulldog breed as it currently stands. Their suggestion is that immediate redefinition of the current breed conformation should be considered.

Both of these studies analysed data from 2016. As more recently registered cohorts of dogs of this and other brachycephalic breeds grow older, the prevalence of these associated conditions will only increase. Using data from an established database of insured dogs in Sweden (2011–16), from which many refereed papers have been published, it is also clear that some brachycephalic breeds are at increased risk of spinal problems than compared to other breeds and may have lower median ages at death [5]. The critical issue now is how such clear breed-related health issues should be approached by owners, breed clubs and breeders to minimise health problems and maximise dog welfare whilst retaining breed attributes and qualities. Where substantive changes have not been forthcoming from the pedigree world, legislation and legal means are increasingly being used to address these issues.

Many stakeholders are involved with dog health and welfare, and are dealing with these various issues and conditions in brachycephalic dogs. Important amongst them is the veterinary profession. In discussion with a veterinary colleague, who specialises in reproduction and is well-known for assisting pedigree dogs, e.g. with artificial insemination (AI) and assisted whelping/caesarean sections, she informed me that approximately 40% of her practice related to brachycephalic dogs. This has prompted a long discussion relating to the ethics of assisting dogs that, without AI and/or surgical intervention, would be unable to reproduce and what could be done to move towards a more ethically conscious practice [6].

The basic tenets of theriogenology /reproductive veterinary practice would suggest that only healthy dogs should be used in assisted breeding. However this aspect has been previously limited and cursory, focussing on perhaps just one of the dogs. But is that enough? Good breeding practices now include various tests to determine the suitability for pairs of mating animals. For example:

- Freedom from obvious signs of disease, including skin problems/allergies.
- Testing for breed-relevant genetic conditions and only mating carriers of deleterious genes to dogs known to be free of the mutation [6].
- Screening for hip/elbow dysplasia, heart conditions and/or hereditary eye diseases as appropriate for the breed.
- Other breed-specific issues.

Veterinarians may have advised on some of these items in the past. However with changing societal expectations and evolving medical knowledge, perhaps it is time that veterinarians should question whether they have been complicit in furthering breed-specific problems and in producing health compromised puppies. An important question for veterinarians is to ask what responsibility they have as a health professional.

After further discussion with my colleague about health conditions in brachycephalic dogs, we recognised that the problems of inherited conditions went further than those covered by the tests listed above (see multiple chapters in [7]). To protect the health and welfare of offspring, it would be logical to exclude dogs from breeding if they have extremes of conformation, including:

- Signs of BOAS (Brachycephalic Obstructive Syndrome) including obvious breathing sounds, as well as,
- Severe skin folds, especially around the eyes, evidence or history of corneal ulcers, or related eye problems.
- Given what we now know about the spinal morphology of these dogs – shouldn’t an x-ray be done to make sure the dog is free of abnormal or missing vertebrae?
- Teeth – we know that breeding, e.g., two of the current style of most brachycephalic dogs will result in puppies with (severely) abnormal teeth.
- And as a basic consideration – should you help to breed a dog that either cannot mate on its own, or where it is likely that it will need surgery in order to whelp?

Without these considerations, are veterinarians normalizing health issues in these breeds? And if they ignore them, what is their motivation? Are they prioritizing what owners want over the welfare and health concerns for the dog, the offspring, and the long-term health of a breed? These are challenging concerns for veterinarians in context of the oath made to protect the well-being of their patients. A concern previously raised by my colleague was that if she followed these guidelines, she might lose up to 40% of her practice. Two years later and her practice has significantly changed. The practice does more in terms of pre-breeding assessments and refuses to assist in breeding dogs affected with deleterious conditions. This has had a considerable impact on income, but as she said, *“Once I saw the problem, I couldn’t ‘un-see’ it”* [6].

Veterinarians do not want to judge their clients. They respect owners’ love for these dogs/breeds, but in the end, professional ethics must prevail. This is for the well-being of dogs and of the veterinary team who treat them and because it is both ethically right and (increasingly) legally correct. Breeders need to understand the seriousness of this challenge for veterinarians and not interpret this as being based on any ‘anti-breed’ sentiments. We need to remind ourselves that societal morals and ethics do change over time as do our tolerance for actions based on personal morals and ethics. A further and more detailed discussion with examples and links relevant to veterinarians and breeders, health and welfare, in this context is available elsewhere [6].

Many countries now have welfare guidelines stating that dogs exhibiting deleterious heritable conditions that could adversely impact on the welfare of the progeny must not be used for breeding. Many cynological organizations have similar statements. For example the Fédération Cynologique Internationale states “Only functionally and clinically healthy dogs, with breed typical conformation, should be used for breeding, i.e. to only use dogs that do not suffer from any serious disease or functional disabilities” (http://www.fci.be/en/Breeding-42.html)). Presumably, a problem arises when ‘breed typical conformation’ includes aspects that are directly related to functional disability and serious disease. The extent to which guidelines are followed or enforced is generally unknown or poor.

The time has come to examine all breeds and dogs in the light of current science, morals, and ethics; to look at aspects of conformation, genetics (e.g. coefficients of inbreeding), health and welfare and ensure that future generations of dogs will be healthier and have better welfare. Any approach taken should not be about vilifying the past. Progress can only be achieved if we are armed with the right scientific evidence – together with a healthy dose of common sense—and if kennel and breed clubs work together and collectively and collaboratively with veterinarians, researchers, and legislators.aa
